# Amyloid Precursor Protein Mediated Changes in Intestinal Epithelial Phenotype *In Vitro*

**DOI:** 10.1371/journal.pone.0119534

**Published:** 2015-03-05

**Authors:** Kendra L. Puig, Gunjan D. Manocha, Colin K. Combs

**Affiliations:** Department of Basic Sciences, University of North Dakota School of Medicine and Health Sciences, Grand Forks, North Dakota, United States of America; INSERM U894, FRANCE

## Abstract

**Background:**

Although APP and its proteolytic metabolites have been well examined in the central nervous system, there remains limited information of their functions outside of the brain. For example, amyloid precursor protein (APP) and amyloid beta (Aβ) immunoreactivity have both been demonstrated in intestinal epithelial cells. Based upon the critical role of these cells in absorption and secretion, we sought to determine whether APP or its metabolite amyloid β (Aβ), had a definable function in these cells.

**Methodology/Principal Findings:**

The human colonic epithelial cell line, Caco-2 cells, were cultured to examine APP expression and Aβ secretion, uptake, and stimulation. Similar to human colonic epithelium stains, Caco-2 cells expressed APP. They also secreted Aβ 1-40 and Aβ 1-42, with LPS stimulating higher concentrations of Aβ 1-40 secretion. The cells also responded to Aβ 1-40 stimulation by increasing IL-6 cytokine secretion and decreasing cholesterol uptake. Conversely, stimulation with a sAPP-derived peptide increased cholesterol uptake. APP was associated with CD36 but not FATP4 in co-IP pull down experiments from the Caco-2 cells. Moreover, stimulation of APP with an agonist antibody acutely decreased CD36-mediated cholesterol uptake.

**Conclusions/Significance:**

APP exists as part of a multi-protein complex with CD36 in human colonic epithelial cells where its proteolytic fragments have complex, reciprocal roles in regulating cholesterol uptake. A biologically active peptide fragment from the N-terminal derived, sAPP, potentiated cholesterol uptake while the β secretase generated product, Aβ1-40, attenuated it. These data suggest that APP is important in regulating intestinal cholesterol uptake in a fashion dependent upon specific proteolytic pathways. Moreover, this biology may be applicable to cells beyond the gastrointestinal tract.

## Introduction

The high numbers of elderly, 30–40%, who experience increased constipation with age may be suffering from a decrease in myenteric acetycholine levels that normally occur with age [1]. Because elderly, including Alzheimer’s disease (AD) patients [2–3], often experience gastrointestinal dysfunction it is reasonable to assume that it may not be a coincidence that weight loss is closely linked, and likely a consequence of Alzheimer’s disease [4]. Unfortunately, the cause of the weight loss remains unclear in addition to whether it has any involvement in disease progression [5]. Prior studies of AD intestines have documented no robust differences from matched controls [6–8]. However, it is clear that amyloid deposits can be observed in human intestine as evidenced by early work examining AD intestines demonstrating amorphous immunostaining in a vascular locus [9]. On the other hand, an immunostaining study of AD intestines ranging from the esophagus to the rectum demonstrated no tangle-like pathology within enteric plexus neurons as assessed by Alz 50 immunoreactivity [8]. Nevertheless, it is difficult to predict the extent of histologic changes in the AD intestines without careful study of all the most relevant AD-related biology in this organ.

For example, although amyloid precursor protein (APP) has been extensively characterized in the central nervous system due to its high level of neuronal expression, there is also abundant evidence from both human and rodent models of Alzheimer’s disease that APP is expressed in the enteric nervous system of the gastrointestinal tract. It is clear that AD human enteric neurons express APP and in some instances demonstrate Aβ plaque-like deposits [6–7–9]. Importantly, although transgenic rodent models of disease also express mutant APP in enteric neurons they present with differences in gastrointestinal disease phenotype [10]. For instance, a prior study demonstrated that the TgCRND8 line [11], expressing human Swedish and Indiana mutation APP under control of the hamster prion promoter demonstrate higher levels of intestinal APP transgene expression compared to the Thy1-hAPP751[12] and APP23 [13] lines. In fact over-expression in this line was even higher in the gut than brain. Not surprisingly, enteric neuron density in the TgCRND8 line was decreased compared to wild type mice and correlated with altered macrophage morphology, decreased motility, and increased TLR4 levels [10]. Collectively, these findings suggest that APP and its metabolites may have some role in intestinal pathology broadly analogous to what occurs in diseased brains.

However, we have shown in our prior work that APP is also robustly expressed in intestinal epithelium in mice [14]. Others have demonstrated that APP and Aβ levels are increased in absorptive columnar epithelial cells in mice fed a high fat diet that is enriched in saturated fat and cholesterol. However, these Aβ levels are attenuated by fasting for 65 h, suggesting that APP or its metabolites may regulate chylomicron biosynthesis [15]. Aβ immunoreactivity colocalizes with apoB in small intestine enterocytes along the lengths of the villi and Aβ levels are attenuated in mice fed a diet free of saturated fat but supplemented with cholesterol, again supporting the idea that Aβ is involved in chylomicron biosynthesis [16–17]. Enterocyte Aβ immunostaining localizes to perinuclear regions suggesting a location within the golgi apparatus or rough endoplasmic reticulum [15]. Although Apo B is typically not considered a brain apolipoprotein, prior work using C57BL/6 mice has demonstrated that mice fed a diet high in palmitic acid demonstrate increased Apo B in the brains of wild type mice which correlated with increased plaque associated Apo B immunoreactivity in an APP/PS1 transgenic line [18]. This suggests that intestinal derived Apo B-Aβ containing lipoproteins might traffic Aβ directly to the brain. Indeed it has been shown in C57BL/6 mice that Aβ co-localizes with intestinal-derived lipoproteins [19]. Perhaps even more interesting is the fact that the TgCRND8 line demonstrates highly elevated plasma Aβ levels and increased very low density lipoprotein triglyceride levels prior to significant brain deposition of plaques [20]. This further suggests that Aβ-mediated changes in lipid metabolism may affect brain deposition [20].

These findings indicate that APP expression and metabolite generation are not limited to a particular cell type in the digestive system with a myriad set of functions attributed to this protein and its metabolites ranging from specific absorption and gut motility to immune response. Since neuronal secretion of Aβ has been well described, in this study we chose instead to focus on the possibility that enterocyte APP metabolism is involved in regulation of their phenotype using the human colorectal adenocarcinoma epithelial cell line, Caco-2.

## Materials and Methods

### Antibodies and Reagents

Anti-occludin and APP antibodies was purchased from Zymed Laboratories (San Francisco, CA). Anti-rabbit (goat), anti-goat (bovine), anti-rat (goat), and anti-mouse (bovine) horseradish peroxidase-conjugated secondary antibodies were purchased from Santa Cruz Biotechnology (Santa Cruz, CA, USA). CD36, FATP4 and GAPDH antibodies were purchased from Santa Cruz Biotechnology (Santa Cruz, CA, USA). Anti-pSrc and Src antibodies were purchased from Cell Signaling Technology Inc (Danvers, MA, USA). Anti-APP (Y188) and LFABP antibodies were purchased from abcam (Cambridge, MA, USA). Aβ 1–40 and 1–42 were purchased from rPeptide (Bogart, GA, USA). The sAPP derived peptide, Arg-Glu-Arg-Met-Ser, was purchased from American Peptide Company Inc. (Sunnyvale, CA, USA). Isotype negative control IgG_1,_ APP agonist antibody 22C11 and SMΦ (CD36 agonist antibody) were purchased from Millipore (Billerica, Massachusetts USA).

### Human Tissue

Human normal adult colon frozen tissue sections were purchased from BioChain Institute, Inc. (Newark, CA, USA). Tissue was immunostained with anti-APP (Y188) or respective secondary only antibodies. Slides were antigen retrieved in boiling Tris-EDTA, pH9, for 20min. Antibody binding in the intestine was visualized using the Vector VIP chromogen (Vector Laboratories, Burlingame, CA). Images were taken using an upright Leica DM1000 microscope and Leica DF320 digital camera system. Figures were made using Adobe Photoshop software (Adobe Systems, San Jose, CA).

### Caco-2 Cells

Caco-2 cells were purchased from ATCC (Manassas, VA, USA). These are an epithelial-like cell line derived from a colorectal adenocarcinoma from a 72 year old male. Caco-2 cells were maintained in DMEM/F12 (Gibco, Life Technologies, Grand Island, NY, USA) supplemented with 10% heat inactivated fetal bovine serum (FBS), 5% donor horse serum (Serum Source International, Charlotte, NC, USA) and antibiotics (0.05mg penicillin/0.05mg streptomycin/0.01mg neomycin/mL) (Sigma-Aldrich, St. Louis, MO, USA). Caco-2 cells were plated and allowed to grow to confluency before removing supplemented media and replacing with serum free DMEM/F12 with or without treatments. Following stimulation, Caco-2 cells were used for ELISA, Aβ uptake, cholesterol assays, immunoprecipitation, and western blot analysis.

### Enzyme-Linked Immunosorbent Assay (ELISA)

Caco-2 cells were incubated overnight in serum free DMEM/F12 with or without 10ng/mL LPS, 100nM Aβ 1–40, 1μM Aβ 1–40, 5μM Aβ1–40, isotype negative control 1μg/mL IgG_1_, or APP agonist antibody 1μg/mL 22C11. The media was collected for Aβ 1–40 and Aβ 1–42 (Invitrogen, Life Technologies, Grand Island, NY, USA), IL-8, MCP-1, MDC, IL-6, and TNFα ELISAs (R&D systems, Minneapolis, MN, USA). The cells were lysed using ice cold RIPA buffer with protease inhibitors and 50U/mL DNAse1. To remove insoluble material, cell lysates were sonicated and centrifuged (14,000 rpm, 4°C, 10 min). The Bradford method [21] was used to quantify protein concentrations for normalization of Aβ in the media and lysates were used for western blotting.

### Aβ Uptake Assay

FITC labeled Aβ 1–40 was prepared according to the manufacturer’s protocol (rPeptide, Bogart, GA, USA). 500nM of the peptide was added to confluent cells in serum free DMEM/F12 for 4 hours in the absence or presence of 10ng/mL LPS. The media was then removed and extracellular peptide signal was quenched by rinsing cells with 0.25% trypan blue dissolved in PBS. The trypan blue incubation served to quench FITC-Aβ signal from any peptide that was associated with the extracellular plasma membrane surface or with the tissue culture plastic of the wells. Intracellular Aβ signal was then quantified using a fluorescent plate reader (480 nm excitation and 520 nm emission).

### Western Blotting

Caco-2 cells were incubated overnight in serum free DMEM/F12 with or without 1μg/mL IgG_1_ (isotype control), 1μg/mL 22C11 (APP agonist antibody), 1μg/mL SMΦ (CD36 agonist antibody), 1μM Aβ 1–40 or both 22C11 and SMΦ. Following stimulation, cells were lysed using ice cold radioimmunoprecipitation assay (RIPA) buffer (20mM Tris, pH 7.4, 150mM NaCl, 1mM Na_3_VO_4_, 10mM NaF, 1mM EDTA, 1mM EGTA, 0.2mM phenylmethylsulfonyl fluoride, 1% Triton, 0.1% SDS, and 0.5% deoxycholate) with protease inhibitors (AEBSF 1mM, Aprotinin 0.8μM, Leupeptin 21μM, Bestatin 36μM, Pepstatin A 15μM, E-64 14μM) and 50U/mL DNAse1(Amresco Inc, Solon, OH, USA). To remove insoluble material cell lysates were sonicated and centrifuged (14,000 rpm, 4°C, 10 min). The Bradford method [21] was used to quantify protein concentrations. Proteins were resolved by 7 or 10% sodium dodecyl sulfate-polyacrylamide gel electrophoresis (SDS-PAGE) and transferred to polyvinylidene difluoride membranes (PVDF) for western blotting using anti-APP (Y188), CD36, occludin, LFABP, FATP4, pSrc, cSrc, and GAPDH (loading control) antibodies. Antibody binding was detected with enhanced chemiluminescence (GE Healthcare, Piscataway, NJ). In some instances, blots were stripped in 0.2 NaOH, 10 min, 25°C, for reprobing. Western blots were quantified using Adobe Photoshop software. Optical densities of bands were normalized against their respective loading controls and averaged (+/-SD).

### Immunoprecipitation

For immunoprecipitation, cells were stimulated for 10 minutes with or without isotype negative control (1μg/mL) IgG_1_, APP agonist antibody (1μg/mL) 22C11, CD36 agonist antibody (1μg/mL) SMΦ, or media alone. Cells were collected and lysed in ice-cold Triton lysis buffer (20mM Tris, pH 7.4, 150mM NaCl, 1mM Na3VO4 10mM NaF, 1mM EDTA, 1mM EGTA, 0.2mM phenylmethylsulfonyl fluoride, and 1% Triton X-100). Cells were homogenized using a teflon pestle. Homogenates were incubated on ice with periodic vortexing for 15 min followed by centrifugation to remove insoluble material (14,000 rpm, 4°C, 10 min). Homogenates were incubated with precipitating antibody (anti-APP) (1 μg of antibody/mg protein lysate) overnight at 4°C, followed by incubation with protein A/G agarose beads (Santa Cruz Biotech, Santa Cruz, CA) for 2 h at 4°C. Resulting immunoprecipitates were washed three times in Triton buffer and resolved by 10% SDS-PAGE and western blotted as described.

### Cholesterol assays

Cholesterol was measured following the protocol provided in the Cholesterol Uptake Cell-Based Assay Kit from Cayman Chemical Company (Ann Arbor, Michigan USA). Briefly, Caco-2 cells were incubated for 40 minutes in glucose free HBSS containing 20μg/ml NBD Cholesterol with or without 10ng/mL LPS, 100nM Aβ 1–40, 1μM Aβ 1–40, 5μM Aβ1–40, isotype negative control (1μg/mL) IgG_1_, APP agonist antibody (1μg/mL) 22C11, CD36 agonist antibody (1μg/mL) SMΦ, both 22C11 and SMΦ, 100pM sAPP peptide, 10pM sAPP peptide, 1nM sAPP peptide, or media alone. At the end of treatment, the plates were aspirated and cell-based assay buffer was added to each well and NBD Cholesterol uptake was quantified using a fluorescent plate reader (480 nm excitation and 520 nm emission).

### Statistical Analysis

The data were analyzed by unpaired two-tailed t-test with or without Welch correction for unequal variance as required, by one-way ANOVA with Holm-Sidak post hoc test.

### Ethic Statement

Use of human tissue samples was approved by the University of North Dakota IRB Committee, protocol number IRB-200412–198.

## Results

### Human colon epithelium demonstrated robust APP immunoreactivity

**Fig 1:**
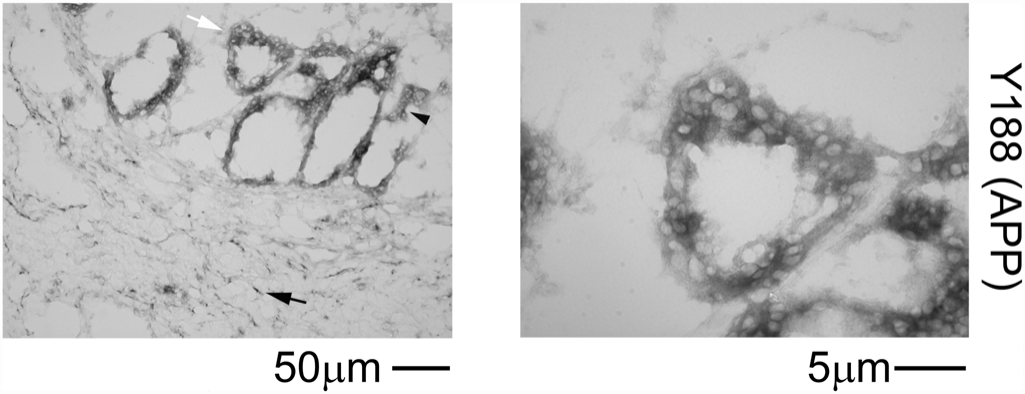
APP is expressed in the human colonic epithelium. Human colon sections were immunostained using anti-APP (Y188) antibody and antibody binding was visualized using Vector VIP as the chromogen. A representative image is shown with a white arrow indicating the region taken for a higher magnification image to demonstrate the epithelial layer shown on the right. Positive staining in the epithelial layer is indicated by a black arrowhead. Positive submucosal staining is indicated by a black arrow.

We previously demonstrated robust APP immunoreactivity within enterocytes and neurons and diffuse immunoreactivity within the smooth muscle of the muscularis externa of the ileum of C57BL/6 mice [14]. In order to validate similar expression of epithelial APP in human intestines, colon sections were immunostained with anti-APP Y188 antibody. Similar to the murine findings, APP localization in the human colon appeared to be in both the epithelial (black arrowhead) and submucosal (black arrow) layers (Fig. 1).

### Human intestinal epithelial cells, Caco-2, secreted Aβ

**Fig 2:**
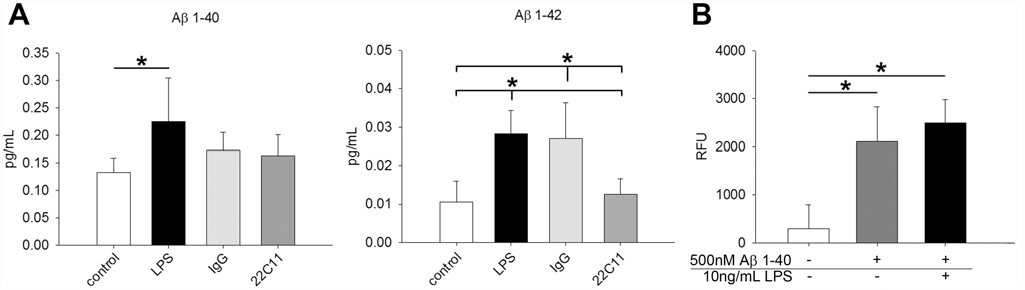
Caco-2 cells secreted and took up Aβ. To demonstrate Caco-2 cell ability to secrete Aβ they were stimulated with or without 10ng/mL LPS, 1μg/mL IgG_1_, or 1μg/mL 22C11 overnight, and Aβ secretion was measured via Aβ 1–40 and Aβ 1–42 ELISA (**A**). To demonstrate Caco-2 cell ability to take up Aβ, they were incubated with or without 500nM FITC conjugated Aβ 1–40 in the absence or presence of 10 ng/mL LPS stimulation for 4 hr. The cells were rinsed with trypan blue to quench extracellular signal from FITC-Aβ on the cell surface or surface of the tissue culture dish and intracellular Aβ fluorescence was measured with a fluorescent plate reader (480 nm excitation and 520 nm emission) (**B**). Data are from 3 experiments in a replicate of 8 each displayed as mean (+/-SD), *p<0.05.

In order to establish an *in vitro* model system that would allow examination of the function of APP in intestinal epithelial cells, the human colonic epithelial-like cell line, Caco-2, was cultured. Endogenous LPS exists in the intestinal lumen as a consequence of the presence of microflora. A variety of studies have documented the effects of this LPS on epithelial cell phenotype and resultant effects on not only the intestine itself but diverse organs including the brain [22–28]. Therefore, the cultures were stimulated with and without a luminal relevant ligand, the bacterial endotoxin, lipopolysaccharide (LPS). Caco-2 cells basally secreted both Aβ 1–40 and Aβ 1–42, with higher concentrations of Aβ 1–40 compared to Aβ 1–42 released upon overnight stimulation with LPS (Fig. 2A). Secretion of Aβ, however, was not increased by stimulating APP overnight with the agonist antibody, 22C11 (Fig. 2A). Since Aβ 1–40 was the more abundantly secreted form of the peptide, we also examined whether Caco-2 cells could be stimulated by the peptide, as might happen in an autocrine fashion. To begin examining this, Caco-2 cells were incubated with fluorescently labeled Aβ 1–40 for 4hr, again in the absence or presence of the luminal ligand, LPS. Interestingly, Caco-2 cells could take up Aβ peptide, although this was not affected by the presence of LPS (Fig. 2B).

### Caco-2 cells were responsive to Aβ stimulation

**Fig 3:**
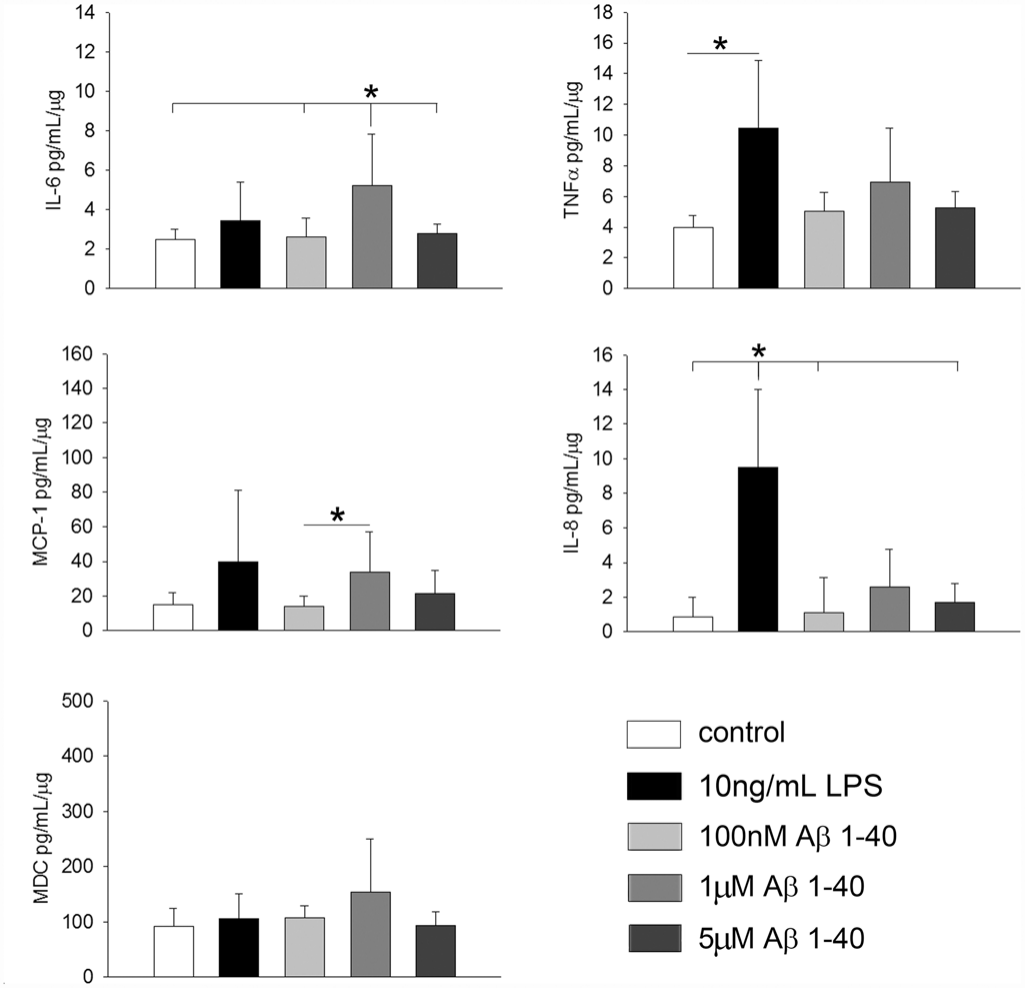
Caco-2 cells secreted increased IL-6 levels upon Aβ stimulation. To demonstrate treatment effects on cytokine secretion cells were stimulated with or without 10ng/mL LPS, 100nM Aβ 1–40, 1μM Aβ 1–40, or 5μM Aβ1–40 overnight and media was analyzed via IL-8, MCP-1, MDC, IL-6 and TNFα ELISAs. Cytokine concentrations are normalized to their respective well protein concentrations (pg/mL cytokine/μg protein) from 6 samples in each condition and are displayed as mean (+/-SD), *p<0.05. Representative data from 3 independent experiments are shown.

To determine whether the Aβ stimulation altered Caco-2 phenotype, we next stimulated cells with increasing concentrations (100nM, 1μM, 5μM) of Aβ 1–40 or 10ng/mL LPS for 24h. ELISA analysis from collected media showed that LPS stimulation increased both IL-8 and TNFα cytokine secretion and 1μM Aβ 1–40 increased IL-6 cytokine secretion by the Caco-2 cells (Fig. 3). These results indicated that enterocytes have the potential to not only secrete Aβ but also respond to extracellular Aβ by a proinflammatory change.

### Aβ and sAPP peptide had reciprocal roles in regulating cholesterol uptake in Caco-2 cells

**Fig 4:**
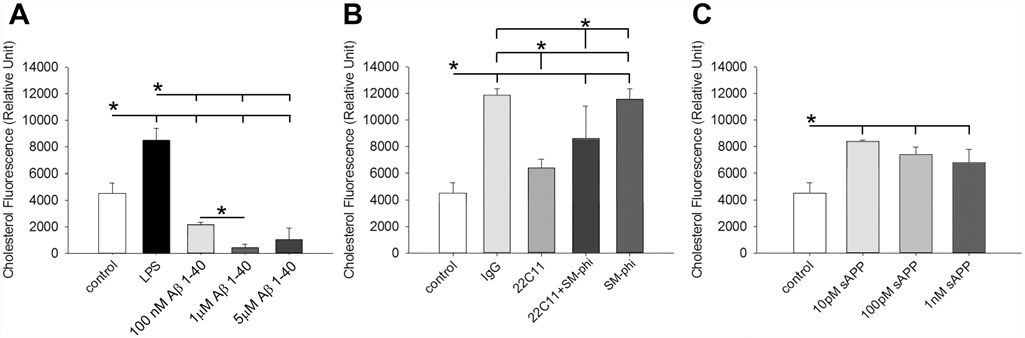
Aβ and 22C11 agonist decreased cholesterol uptake whereas the sAPP-derived peptide and SM-ϕ stimulated increased cholesterol uptake by Caco-2 cells. To demonstrate effects on lipid uptake a cell-based cholesterol uptake assay was performed. Cells were stimulated with or without 10ng/mL LPS, 100nM Aβ 1–40, 1μM Aβ 1–40, 5μM Aβ1–40, 1μg/mL IgG_1_ (isotype control), 1μg/mL 22C11 (APP agonist), 1μg/mL SMΦ (CD36 agonist), 10pM, 100pM, and 1nM sAPP peptide, or both 22C11 and SMΦ for 40 min and NBD Cholesterol uptake was quantified using a fluorescent plate reader (480 nm excitation and 520 nm emission). Data are from 8 samples in each condition and are displayed as mean (+/-SD), *p<0.05. Representative data from 3 independent experiments are shown.

It has been shown that APP and Aβ levels are increased in absorptive columnar epithelial cells in mice fed a high fat diet enriched in saturated fat and cholesterol suggesting some role in chylomicron formation [15]. To further examine a role for APP and its metabolites in regulating lipid uptake we used the Caco-2 cell cultures to quantify changes in cholesterol uptake. Cells were stimulated with increasing concentrations of 100nM, 1μM and 5μM Aβ 1–40 or 10ng/mL LPS for 40 min. LPS stimulation increased cholesterol uptake while all three concentrations of Aβ decreased cholesterol uptake with the most dramatic effect at 1μM Aβ 1–40 (Fig. 4A). Although the APP agonist antibody, 22C11, did not alter Aβ secretion, we determined whether 22C11 or SM-ϕ, CD36/fatty acid translocase agonist antibody would alter cholesterol uptake in the cells. Cells were stimulated for 40 min with isotype control IgG, 22C11, SM-ϕ, or both 22C11 and SM-ϕ. IgG, SM-ϕ and the combination of 22C11 and SM-ϕ all increased cholesterol uptake compared to untreated control cells (Fig. 4B). 22C11 decreased cholesterol uptake compared to its isotype control, IgG, as well as from SM-ϕ alone (Fig. 4B). To determine whether another proteolytic fragment of APP could alter cholesterol uptake, a growth promoting fragment of the N-terminal secreted APP (sAPP) [29], was used in increasing concentrations (1nM, 10pM, 100pM) to stimulate the Caco-2 cells for 40 min. In contrast to Aβ 1–40, cholesterol uptake was increased by all three concentrations of sAPP peptide with no dose-dependent differences (Fig. 4C).

### APP, CD36, or Aβ Stimulation of Caco-2 cells selectively altered protein levels

**Fig 5:**
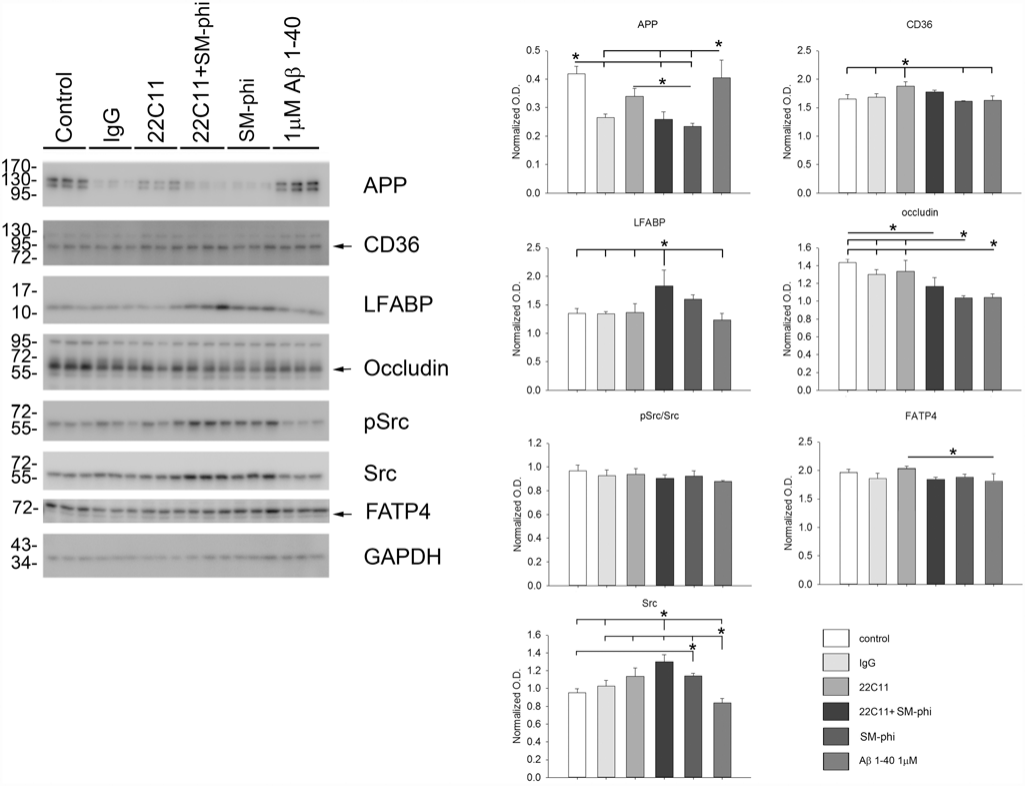
Aβ, APP, and CD36 stimulations selectively altered protein levels in Caco-2 cells. To assess changes in phenotype Caco-2 cells were stimulated overnight with or without 1μM Aβ 1–40, 1μg/mL IgG_1_ (isotype control), 1μg/mL 22C11 (APP agonist), 1μg/mL SMΦ (CD36 agonist), or both 22C11 and CD36 and then lysed for western blotting. Data are from 3 samples in each condition and are displayed as mean (+/-SD), *p<0.05.

Based upon the Aβ-mediated changes in cholesterol uptake and cytokine secretion observed, protein levels of enterocyte relative proteins were next examined to better determine changes in cellular phenotype following stimulation. Caco-2 cells were stimulated overnight with the isotype control IgG, 22C11, SM-ϕ, both 22C11 and SM-ϕ, or 1μM Aβ 1–40. CD36 stimulation decreased both APP and the tight junction marker, occludin, protein levels with a slight but not quite significant increase in fatty acid binding protein, LFABP, and no effect on protein levels of fatty acid translocase/CD36, or the fatty acid transport protein, FATP4. Although CD36 stimulation alone or in combination with 22C11 agonist antibody did not increase levels of active, pSrc, both stimulated a surprising, significant increase in total Src kinase levels (Fig. 5). APP stimulation with 22C11 agonist antibody increased CD36 with no effect on APP, LFABP, occludin, FATP4, or active pSrc protein levels (Fig. 5). Interestingly, combined CD36 and APP stimulation increased LFABP protein levels (Fig. 5). 1μM Aβ 1–40 stimulation decreased occludin protein levels compared to control untreated cells with no effect on other proteins examined (Fig. 5).

### APP was associated with CD36 in Caco-2 cells

**Fig 6:**
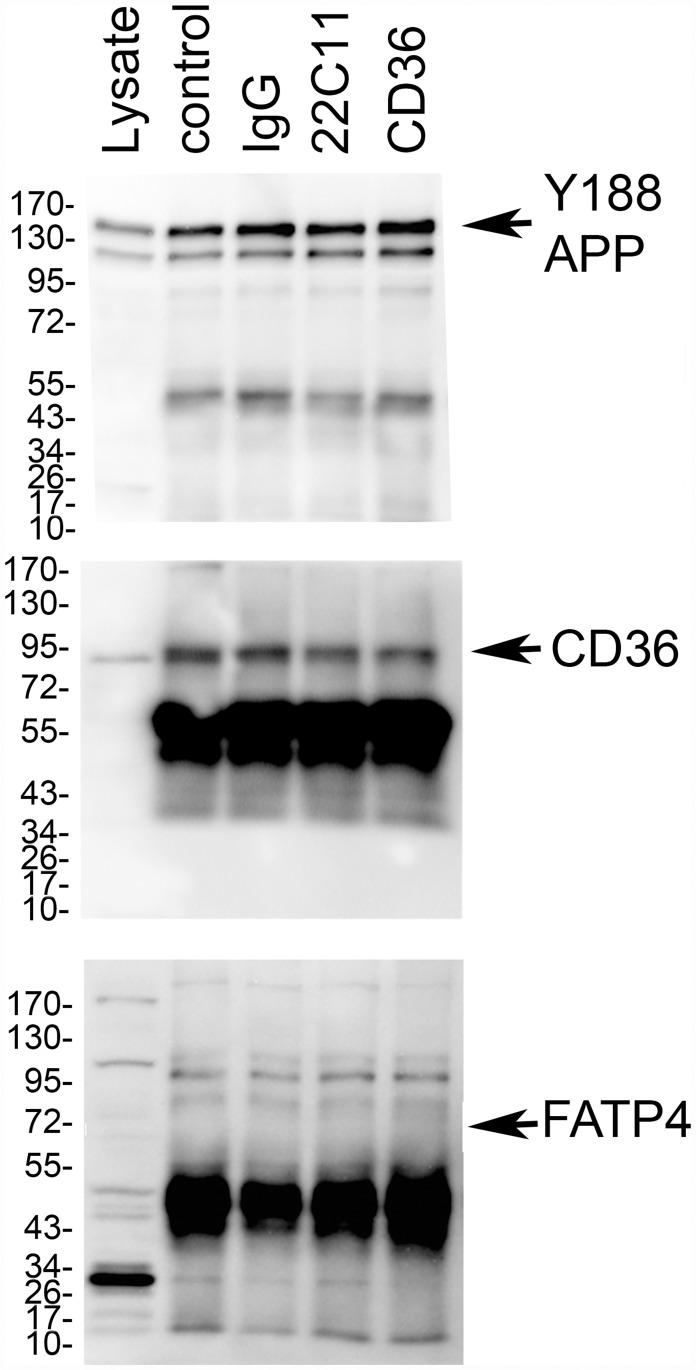
APP was associated with CD36 in Caco-2 cells. To explore whether APP was part of a multi-protein complex with CD36, co-immunoprecipitation pull-down experiments from cultured Caco-2 cells were performed. Cells were stimulated with or without 1μg/mL IgG_1_ (isotype control), 1μg/mL 22C11 (APP agonist), 1μg/mL SMΦ (CD36 agonist) for 10 min. and then homogenized and immunoprecipitated with precipitating antibody (anti-APP) (1 μg of antibody/mg protein lysate). Resulting immunoprecipitates were resolved by 10% SDS-PAGE and western blotted using anti-APP (Y188), CD36, or FATP4 antibodies. Data shown are representative of three independent experiments.

Since APP stimulation moderately increased CD36 protein levels and CD36 stimulation decreased APP protein levels, we hypothesized that these two proteins might exist as components of a multi-protein complex. Co-immunoprecipitation pull-down experiments from 10 min stimulated Caco-2 cells were performed. Immunoprecipitation with anti-APP antibody verified that CD36 is complexed with APP in the Caco-2 cells basally. Moreover, 10 min pre-stimulation with the APP agonist, 22C11, and the CD36 agonist, SM-ϕ, did not appear to promote or attenuate APP-CD36 association (Fig. 6). This association appeared somewhat selective since an additional intestine relevant fatty acid transport protein, FATP4, did not co-immunoprecipitate with APP (Fig. 6).

## Discussion

Human colon epithelium demonstrated robust APP immunoreactivity similar to what has been observed by our group and others suggesting that APP and its metabolites have a function in the gastrointestinal tract. Our data demonstrated that Caco-2 cells secreted both Aβ 1–40 and Aβ 1–42, with higher quantifiable amounts of Aβ 1–40 compared to Aβ 1–42 upon stimulation with LPS. These enterocytes can also take up Aβ 1–40 peptides and stimulate in an autocrine fashion to increase IL-6 cytokine secretion. More importantly, stimulation of the Caco-2 cells with APP cross-linking antibody or Aβ attenuated the ability of the cells to take up cholesterol. On the other hand, sAPP-derived peptide stimulation increased cholesterol uptake. Clearly, future work is needed to determine whether altered levels of cholesterol or specific fatty acids may affect the ability of APP to regulate not only cholesterol uptake but possibly individual fatty acid uptake as well. This demonstrated a complex role for APP and its metabolites in regulating cholesterol absorption and was entirely consistent with the fact that APP was part of a multi-protein complex with CD36 in the Caco-2 cells.

One exciting observation from our study was that Caco-2 cells can be stimulated to secrete Aβ peptides. Aβ secreted from epithelial cells may be interacting with the immune cells, enteric neurons, and themselves in an autocrine fashion to regulate a complex multi-cellular behavior in the intestines. We observed that the Caco-2 cells were stimulated to increase secretion of Aβ 1–40 and 1–42 by LPS. Surprisingly, however, cross-linking APP to increase APP metabolism to Aβ did not result in increased Aβ secretion consistent with no change in full length APP levels with 22C11 stimulation. One possibility is that the levels of Aβ were simply too low for our ELISA detection. Another, more likely possibility, is that cross-linking of APP in these cells may stimulate an APP-dependent signaling response that does not involve increased processing to Aβ. In addition, it is possible that the agonist antibodies themselves, somehow did not sufficiently serve as full agonists to the Caco-2 cell APP perhaps due to limitations in cell surface localized APP or simply differences in APP function in this cell type. Indeed, at this point, the increased secretion of Aβ that resulted from LPS stimulation may well have been from intracellular APP. Additionally, there is even the possibility that LPS may initiate amyloid synthesis without APP processing. At this point, it is unclear what the consequence of increased Aβ secretion would be *in vivo* and why this would need to increase in response to stimuli such as LPS. Based upon the fact that the Caco-2 cells could take up the peptide through a currently undefined mechanism, as evidenced by FITC-labeled Aβ 1–40 uptake assays, it is intriguing to speculate that the peptide acts in an autocrine fashion after enterocyte interaction with the microflora to regulate their phenotype. For instance, we did observe that Aβ 1–40 stimulated increased IL-6 secretion from the Caco-2 cells. It was interesting to note that only 1μM Aβ 1–40 and not 5μM Aβ 1–40 stimulated IL-6 secretion. Based upon the fact that Aβ peptides can form various multimeric states including fibrils during the 24 hour stimulation we utilized, we have no evidence to support this at the moment but speculate that different conformations of the peptide exist at each concentration thus providing unique stimuli to the cells. The effect of Aβ at 1μM but not 5μM stimulating cytokine secretion may even reflect unique Aβ-receptor interactions. Based upon the higher concentrations of Aβ 1–40 we observed, we elected to focus on examining effects of this shorter peptide. However, we have not ruled out the possibility that Aβ 1–42 may also stimulate the cells, perhaps in a unique fashion from Aβ 1–40. This is particularly relevant when considering the higher propensity for fibrillization of the larger Aβ 1–42 peptide. One can imagine a scenario where shunting of APP processing to primarily Aβ 1–42 peptide production could lead to increased fibrillization of Aβ and likely a different stimulus than nonfibrillar Aβ 1–40 or Aβ 1–42. In addition, should both peptides be present, it is even feasible that unique types of Caco-2 cell interactions could occur involving different multimeric states of either peptides.

Although our study focused on the *in vitro* ability of the cells to take up Aβ, it is clear that Aβ can be taken up by intestinal epithelial cells *in vivo*. A prior study gavaging suckling cows or mice with Aβ-EGFP fusion protein demonstrated that the peptide could reach the ileum and be taken up into villous epithelium cells even reaching the Peyer’s patches and spleen [30–31]. Prior work has shown that amyloidogenic proteins in the intestine are often elevated during acute phase responses or during chronic infection directly influence intestinal immune cell phenotype. For example, it is clear that serum amyloid A stimulation of epithelial cell lines *in vitro* is sufficient to increase their secretion of cytokines and NFκB activity [32]. Secondary amyloidosis in the GI tract is also a common consequence of chronic infection and inflammation [33–34]. Therefore accumulation of Aβ might be directly affecting intestinal immune phenotype.

The mammalian intestines are characterized by an abundance of resident immune cells, including macrophages necessary for monitoring resident and foreign microbial exposure [35]. We have previously demonstrated that mice which lack APP have decreased numbers of macrophage in the ileum [14]. Therefore, it is possible that the proinflammatory environment and microglia/macrophage numbers and activation state in the intestines are to some degree dependent upon APP expression or metabolism. It is well demonstrated that microglia *in vitro* induce the expression of multiple proinflammatory cytokines, chemokines, reactive oxygen species, and nitrogen species in response to Aβ, all of which cause neuronal damage [36–39]. Aβ has been demonstrated to regulate the proinflammatory state of human monocytes/macrophages increasing metalloproteinases, chemokines, cytokines, and reactive oxygen species [40–49]. Based upon these data, it is not unreasonable to assume that the Aβ being secreted from epithelial cells may be interacting with the intestinal immune cells to alter their phenotype.

As expected, stimulation of CD36 with SM-ϕ agonist antibody resulted in increased cholesterol uptake by the Caco-2 cells. Cross-linking APP had no effect on basal cholesterol uptake but, surprisingly, this attenuated the ability of CD36 agonist antibody to stimulate cholesterol uptake. These data demonstrated that APP or its metabolites have a negative effect on absorption and were consistent with the fact that Aβ stimulation of the Caco-2 cells also attenuated cholesterol uptake. Consistent with our work, a prior *in vitro* study using the human HepG2 hepatocyte line demonstrated that Aβ 1–40 treatment is sufficient to attenuate steady state levels of intracellular cholesterol, cholesterol esters, phospholipids, and triacylglycerol [50]. Another study using the human HepG2 cell line demonstrated that these cells can secrete Aβ in association with lipoproteins containing Apo J, ApoA-I, phospholipids, triglycerides, cholesterol and cholesterol esters suggesting a role in lipid transport [51]. Similarly, a study using C57BL/6 mice demonstrated that Aβ is secreted as part of an Apo B containing chylomicron with immunoreactivity co-localizing to intestinal epithelial cells [16] and Aβ co-localizing with intestinal derived lipoproteins [19]. Although we did not examine this, it is quite likely the case that the Caco-2 cells are also able to secrete Aβ as a component of a lipoprotein complex since they are capable, for instance, of both producing and secreting apoB100 and apoB48 [23–29]. High fat feeding also increases Aβ immunoreactivity in small intestinal epithelial cells of the C57BL/6 mice and this effect is potentiated in mice lacking Apo E [34–52]. Moreover, radiolabeled Aβ 1–40 can associate with chylomicron-like particles and when intravenously injected, the peptide can be transported into diverse organs including adipose tissue and brain [53]. Even though Apo B is often not considered a brain apolipoprotein, prior work using C57BL/6 mice has demonstrated that mice fed a diet high in palmitic acid demonstrated increased Apo B in the brains of wild type mice which correlated with increased plaque associated Apo B immunoreactivity in an APP/PS1 transgenic line [18]. Collectively these data suggest not only that intestinal APP and Aβ may be involved in regulating cholesterol absorption from the intestines but also Aβ supply to the brain.

Perhaps not surprisingly, we demonstrate that the parent protein, APP, is part of a multi-protein complex with CD36 in the Caco-2 cells. CD36 is a key fat absorption regulator involved in fatty-acid uptake in these enterocytes [54]. CD36 binds to a host of ligands including anionic phospholipids, collagen, oxidized LDL, thrombospondin-1, β-amyloid, and *Plasmodium falciparum* [55–58]. CD36 facilitates the uptake of carotenoids in Caco-2 cells [59]. CD36 also facilitates free cholesterol uptake [60]. Therefore the mechanisms of interaction between APP and CD36 may warrant further investigation as our data demonstrates that the use of an APP agonist antibody, 22C11, decreases CD36-mediated cholesterol uptake in these Caco-2 cells. These data suggest that a portion of the mechanism by which stimulation with APP cross-linking antibody or Aβ results in attenuated cholesterol uptake is via inhibition of CD36 function.

We have also demonstrated that Aβ decreases occludin levels in these Caco-2 cells suggesting that Aβ is directly involved in regulating tight junction formation. It has already been demonstrated that an Aβ-RAGE interaction exists in which to disrupt blood brain barrier integrity decreasing zonula occludin-1 [61]. This disruption of the intestinal barrier may be consistent with changes seen in blood-brain barrier integrity and may similarly contribute to the neuropathological consequences of Alzheimer’s disease.
